# Individualized total laparoscopic surgery based on 3D remodeling for portal hypertension: A single surgical team experience

**DOI:** 10.3389/fsurg.2022.905385

**Published:** 2022-08-10

**Authors:** Yin Jikai, Wang Dong, Zhang Li, Dong Rui, Yang Tao, Huang Bo, Sun Yibo, Lei Shixiong, Bai Qiangshan, Lu Jianguo

**Affiliations:** ^1^ Department of General Surgery, TangDu Hospital, Fourth Military Medical University, Xi’an, China; ^2^ Department of Ultrasound medicine, TangDu Hospital, Fourth Military Medical University, Xi’an, China

**Keywords:** cirrhosis, portal hypertension, 3D remodeling, laparoscopic splenectomy, splenomegaly

## Abstract

**Background and aims:**

Portal hypertension (PHT) is common in end-stage cirrhosis, and variceal bleeding is the main complication associated with mortality. Surgery is usually performed in patients with PHT with a high risk of variceal bleeding in China. This study aimed to introduce an individualized and precise total laparoscopic surgical procedure based on 3D remodeling for PHT.

**Methods:**

From March 2013 to December 2018, 146 patients with cirrhotic PHT underwent a laparoscopic surgical procedure in our department. An individualized 3D remodeling evaluation was carried out for surgical planning.

**Results:**

The operation time was 319.96 ± 91.53 min. Eight of 146 patients were converted to open surgery. Acute portal vein system thrombosis occurred in 10 patients (6.85%). During the first year, 11 patients (8.15%) experienced rebleeding and two (1.48%) died. 18 patients (13.33%) experienced rebleeding and three died, giving a 3-year mortality rate of 3.66%. Compared with preoperatively, the portal vein showed significant postoperative decreases in diameter, flow velocity, and flow amount, while the hepatic artery showed significant postoperative increases in diameter, flow velocity, and flow amount. A 3D liver volume evaluation found that 19 of 21 patients had a significantly increased liver volume postoperatively, and a significantly decreased MELD score.

**Conclusion:**

This retrospective study introduced a safe, feasible, and effective individualized surgical procedure. Our results show that this surgical treatment may not only act as an effective symptomatic treatment for PHT to prevent esophageal and gastric hemorrhage, but also as an etiological treatment to increase liver function and long-term survival.

## Introduction

Portal hypertension (PHT) is a common and severe syndrome in patients with cirrhosis, especially decompensated cirrhosis. Furthermore, the incidence of PHT is much higher in China than in any other country (1). Gastroesophageal varices and subsequent variceal bleeding are major life-threatening events. Although liver transplantation is considered the only curative treatment for PHT secondary to all end-stage liver diseases, only few patients with PHT actually receive liver transplantation due to ineligibility for transplantation, lack of liver donors, or high medical costs (2). Although standardized strategies based on risk assessment have improved the clinical outcomes of upper gastrointestinal bleeding secondary to PHT, this disease still causes about 150,000 deaths per year in Europe (3). As China has a high incidence of hepatitis infection and liver cirrhosis, PHT is a serious problem.

Non-transplant surgical strategies for PHT such as splenectomy, devascularization, and shunt operations were widely used to prevent variceal bleeding for decades until the transjugular intrahepatic portosystemic shunt (TIPS) became a relatively safe procedure in large medical centers in select patients. TIPS is not recommended for those without bleeding history due to the potential damage to liver function, and is usually applied as a salvage treatment for patients waiting for liver transplantation. However, few patients actually receive liver transplantation, and TIPS shows no survival benefit in the real-world population (4, 5), except in very specific high-risk patients. TIPS and non-selective beta-blockers may offer better short-term survival (6), but recent clinical trials found no long-term survival benefit for patients with PHT who received TIPS or other non-surgical treatments (7). In contrast, a Japanese randomized clinical trial of non-shunting procedures (including splenectomy) reported that surgical treatments may bring significant long-term survival benefits to patients with PHT at high risk of bleeding (8).

Splenectomy plus devascularization is still the most widely used surgical procedure for PHT in China (9). However, due to the complexity of the multiple pathologies accompanying PHT, especially coagulation dysfunction, potential massive intraoperative blood loss is a major concern in any surgery in cirrhotic patients, especially those with PHT. Furthermore, splenomegaly is considered a contraindication for a minimally invasive approach. With the recent development of laparoscopic techniques and instruments, laparoscopic splenectomy and devascularization have proved to be safe, feasible, and minimally invasive procedures for PHT (10). However, the clinical outcomes of surgical treatments vary between medical centers, largely due to individual differences, surgical techniques, and poor understanding of the disease.

Since 2013, our surgical team has used an individualized surgical strategy for patients with PHT. Our department has experience using modified laparoscopic splenectomy plus devascularization, modified laparoscopic splenectomy plus devascularization plus shunt, individualized devascularization, and individualized devascularization plus shunt. Individualized devascularization is the most commonly used procedure, and is based on the individualized assessment of varicose veins and their major drainage veins. This retrospective study focused on the surgical design, safety, effects, and clinical outcomes of this individualized devascularization procedure, and the possible clinical benefits for patients with PHT.

## Methods

### Patients and clinical data

The study population comprised 146 patients with PHT who underwent individualized laparoscopic surgery between March 2013 and December 2018. The general preoperative assessment comprised abdominal ultrasonography, contrast-enhanced computed tomography (CT), blood tests, and other routine biochemistry tests. The liver function was routinely evaluated using the Child-Pugh score and Model for End-stage Liver Disease (MELD) score, and the liver function preserve was assessed using the indocyanine green (ICG) clearance test. Endoscopic examination was routinely performed to evaluate the location and degree of gastroesophageal varices (11). The hepatic venous pressure gradient (HVPG) was measured to assess intrahepatic resistance and bleeding tendency in some patients, especially those without a clear history of bleeding. All patients were followed up at 1, 6, and 12 months postoperatively. Final follow-up data were collected from medical records or by telephone interview. This study was approved by the Ethical Committee of Tangdu Hospital (approval number: 202011-32).

### Surgical inclusion and exclusion criteria

The indications for surgery were: (1) age 18–70 years; (2) gastroesophageal varices identified on endoscopic examination; (3) recurrent gastroesophageal variceal bleeding that could not be controlled by endoscopic treatment; (4) patients with PHT with no clear hemorrhage history, but with large varices (>5 mm), and a HVPG of >12 mmHg, indicating a high risk of bleeding events and death in the near future (12, 13); (5) patients who confirmed that they would not accept and/or could not afford liver transplantation in the near future. The surgical exclusion criteria were based on the following indicators of liver dysfunction: (1) Child-Pugh class B minus (>8 points) or worse; (2) ICG15 of over 40%, uncontrollable moderate to massive ascites; (3) uncontrollable severely prolonged prothrombin time of >18 s; (4) serum bilirubin >51 µmol/L. The hypersplenism and concomitant thrombocytopenia (platelet count <50 × 10^9^/ml) always seen in cirrhotic patients were not considered contraindications to surgery in those with normal bone marrow proliferation ability.

## Preoperative evaluation

### 3D evaluation of the major vein connecting the portal vein and gastroesophageal varices

**Figure 1:**
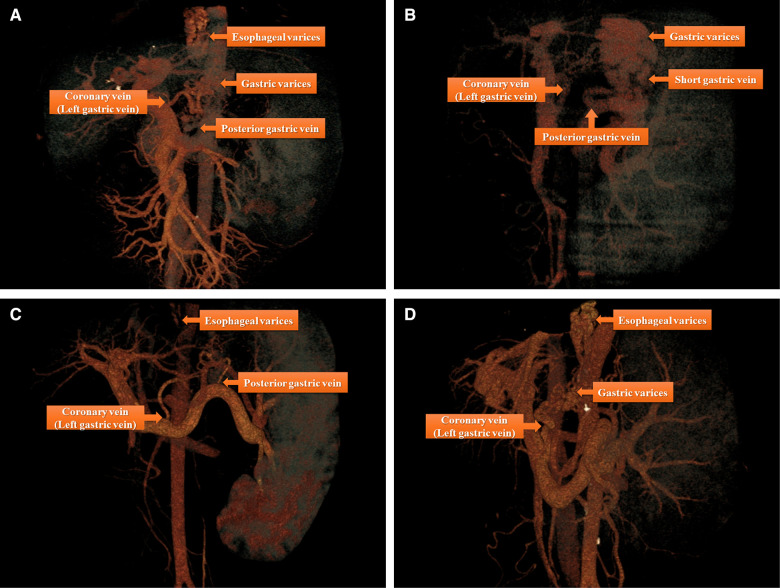
There are four major types of anatomical variations of portal vein system by three-dimensional images reconstruction based on the data derived from 64-slice spiral CT: (**A**) Esophageal and gastric varices (the source of bleeding) were found to flow into coronary vein (left gastric vein) and posterior gastric vein; (**B**) gastric varices (the source of bleeding) were found to flow into posterior gastric vein and short gastric vein; (**C**) Esophageal varices (the source of bleeding) were found to flow into coronary vein (left gastric vein) and posterior gastric vein; (**D**) Esophageal and gastric varices (the source of bleeding) were found to flow into coronary vein (left gastric vein).

The image data of 64-slice spiral CT were analyzed using a RadiAnt DICOM Viewer (free 64-bit version available at https://www.radiantviewer.com/); the software then reconstructed 3D images with vascular strengthening to identify the vein(s) connecting the gastroesophageal varices and portal vein system. The major connecting vein or veins were located for surgical planning as shown in Figure 1.

### Individualized laparoscopic procedures

**Figure 2:**
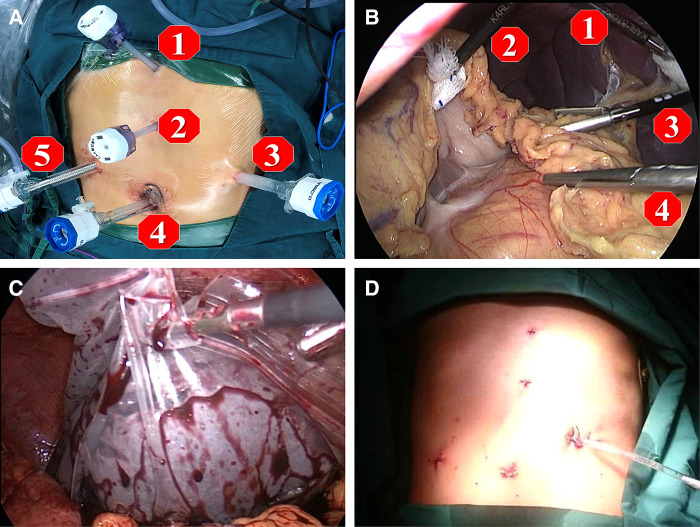
Five ports technique is applied for the operation as shown in figures. (**A,B**) port 1 and 2 are assigned for the first assistant, port 3 and 4 are main operation ports for the operator, and port 5 is the observation port for the second assistant. In order to obtain the best view for operation, the second assistant need to adjust the 30° angle of laparoscope to fit the view of operator; (**C**) at the end of operation, the spleen was placed into a plastic specimen bag, then smashed and removed from the abdominal cavity by enlarging the incision of the 12 mm port 3; (**D**) after surgery, a drainage tube was placed at Spleen Fossa for postoperative observation.

After the induction of general anesthesia, patients were placed in the supine position with split legs. We applied a five-port technique as shown in Figure 2. Three 12-mm trocars were placed; one 12-mm trocar for observation was placed 5 cm higher than the crossing point of the right midclavicular line and a line at the level of the umbilicus, one for the operator was inserted beneath the umbilicus, and another for the operator was placed at the surface projection of the lower edge of the spleen. Two 5-mm ports for the assistant were placed on the line between the xiphoid and umbilicus. After all ports were placed, the 30° reverse Trendelenburg position was applied to achieve better vision intraoperatively.

**Figure 3:**
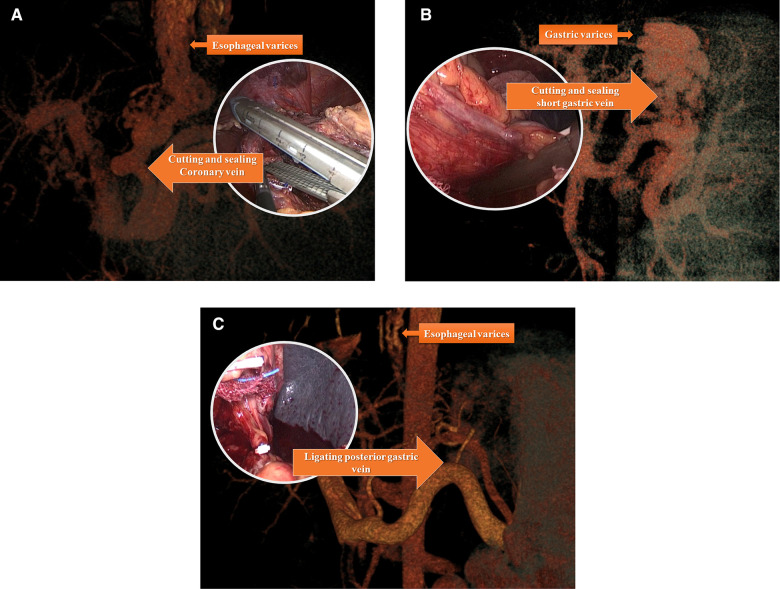
Individualized devascularization procedure usually disconnected major vessel trunk include coronary vein (left gastric vein) (**A**) posterior gastric vein (**B**) and short gastric vein (**C**) individually or in combination according to preoperational vascular assessment.

A LigaSure vessel-sealing device (Covidien, Boulder, CO, USA) was routinely used. The first step was to expose the omental pouch to identify, separate, and ligate the splenic artery at the upper edge of the pancreas. Reliable ligation of the splenic artery was vital to ensure perioperative safety, especially after surgery, due to reduced pressure in the vessels surrounding the spleen and left stomach. Splenectomy and pericardial devascularization were basically performed as described by Hassab (14). However, there were procedural modifications in two major steps: the length of the lower esophagus for devascularization was extended to about 8 cm by pulling down the esophagus from the diaphragmatic hiatus, and an individualized and precise esophagogastric vascular disconnection procedure was performed in accordance with the reconstruction of the major vein(s) connecting the gastroesophageal varices and portal vein system. The vessel trunk of the major connecting vein was sealed using an endoscopic linear cutting stapler or absorbable ligation clip in accordance with the trunk diameter (Figure 3). The LigaSure vessel-sealing device was used to coagulate and cut vessels with a diameter of <5 mm. The individualized procedures varied among patients due to the different combinations of major vessels. Intraoperatively, the pancreatic tail was carefully protected from damage. At the end of surgery, the entire spleen was put into a plastic specimen bag (Figure 2C) and pulled out to the surface of the abdominal wall. The spleen was cut into pieces in the bag and removed by sponge forceps through an enlarged incision from the 12-mm port on the left side. A drainage tube was placed at the splenic fossa for postoperative observation. The patients were encouraged get out of bed activities early and the enoxaparin (0.4 IU twice daily) was used on postoperative 48 h if no obvious abdominal bleeding and the coagulation function was stable. Patients took Rivaroxaban tablets (15 mg /day, Bayer, Germany) for three months for preventing portal vein thrombosis.

### Statistical analysis

Statistical analyses were carried out with the Statistical Package for Social Sciences for Windows (version 20.0; SPSS Inc., Chicago, IL, USA). Continuous variables were described as mean ± standard deviation. Categorical variables and continuous variables with normal distribution were compared by Pearson's *χ*^2^ test and the Student's *t* test, respectively. Data with skewed distribution were described as median ± quartile, and tested with the non-parametric Mann-Whitney U test. *P* < 0.05 was considered to indicate a significant difference.

## Results

### Patient characteristics

All patients received surgical treatments by the same single surgical team. The clinical data are summarized in Table 1. There were 80 men and 66 women, with a mean age of 45.47 ± 10.03 years (range 22–73 years). One-hundred-and-seven patients had a definite history of upper gastrointestinal hemorrhage including hematemesis and/or melena, while the other 39 patients received surgery due to a clinical evaluation suggesting a very high risk of bleeding within 1 year (large gastroesophageal varices >5 mm and HVPG >12 mmHg) (15).

**Table 1:** Patients characteristics (*n* = 146).

characteristics	Value (Range and/or percentage) (*n*)
Age, years(range)	45.47 ± 10.03 (22–73)
Sex	
Male	80(54.8%)
Female	66(45.2%)
BMI	22.36 ± 2.88 (16.66–29.07)
Etiology	
HBV	108(74.0%)
HCV	14 (9.6%)
HBV&HCV	1 (0.7%)
Autoimmune hepatitis	7(4.8%)
Cholestatic Cirrhosis	6(4.1%)
Other	10(6.8%)
Comorbidities	
Diabetes mellitus	10(6.8%)
Hypertension	5(3.4%)
Diabetes mellitus & Hypertension	2(1.4%)
Gastroesophageal varices	
small varices <5 mm	18(12.3%)
large varices >5 mm	128(87.7%)
Red color signs on varices	
Yes	114(78.1%)
No	32(21.9%)
Sarin’s classification	
GOV1	96(65.8%)
GOV2	35(23.9%)
IGV1	7(4.8%)
IGV2	8(5.5%)
Child-Pugh score	6.01 ± 0.80(5–9)
Child-pugh grade	
A	110(75.3%)
B	36(24.7%)
MELD	9.39 ± 2.20(2.58–14.52)
History of bleeding	
Yes	107(73.3%)
No	39(26.7%)
History of abdominal surgery	
Yes	8(5.5%)
No	138(94.5%)
HPVG	15.90 ± 5.53 (2–28) (*n* = 111)
ICG15 (%)	14.27 ± 8.15 (1.7–39.5) (*n* = 108)
The size of spleen	
Thickness (cm)	6.26 ± 1.04 (4.6–8.8) (*n* = 116)
Length (cm)	18.06 ± 2.69(11.4–25.0) (*n* = 88)

The causes of cirrhosis and PHT were viral hepatitis B (*n* = 108) and C (*n* = 14), alcoholic cirrhosis and cholestatic cirrhosis (*n* = 6), autoimmune hepatitis (*n* = 7), and other unidentified causes of cirrhosis (*n* = 10). One patient had both viral hepatitis B and C. All operations were performed as elective operations, and all patients received individualized preoperative evaluations. Liver function assessments revealed Child-Pugh class A in 110 patients, and class B in 36 patients, while the mean ICG15 was 14.27 ± 8.15 (range 1.7–39.5). On preoperative endoscopic examination, the varices were defined as large (≥5 mm) or small (<5 mm) in accordance with a previous report (15). Large varices were identified in 128 patients, while small varices were identified in 18 patients. Red color signs were found in 114 patients.

### Individualized evaluation

The three major portal-systemic collaterals connecting the esophageal and gastric varices to the portal vein were the coronary vein (left gastric vein), posterior gastric vein, and short gastric vein. Theoretically, there are seven kinds of collaterals formed by the following combinations: each of the three veins alone, any two combinations of three veins, or the three veins combined. However, three types of collaterals were commonly identified in the present study (Figure 1). The coronary vein was the most important drainage vessel connecting the portal vein to the varices alone or in combination with other veins; however, in some cases, the coronary vein was not the major vessel responsible for the gastric varices (Figure 1B).

### Perioperative information

Perioperative data are summarized in Table 2. Operation time was defined as the time from the establishment of pneumoperitoneum until the completion of all surgical procedures, before the spleen was removed from the abdominal cavity. Mean operation time was 319.96 ± 91.53 min (range 180–720 min), and mean blood loss was 520.08 ± 606.46 ml (range 20–3,500 ml). There were eight patients converted to open surgery due to uncontrolled massive intraoperative blood loss (>800 ml). Three unplanned reoperations were performed due to postoperative drainage indicating possible active bleeding; the bleeding site was later confirmed to be the operational trocar site of the abdominal wall in all three cases. These adverse events mainly happened in 2013–2014. The major reason for reoperations may have been largely due to undiscovered trocar damage. With the progresses in technical proficiency and standardization of intraoperative management, no unplanned reoperations have been necessary since 2015.

**Table 2:** Perioperative data (*n* = 146).

Variables	Value (Range or pecentage)
Operation time	319.96 ± 91.53(180–720)
Intraoperative blood loss	520.08 ± 606.46(20–3,500)
Transfusions	77(52.7%)
Conversion to open surgery	8(5.5%)
Unplanned reoperation	3(2.1%)
Out of bed activity(days)	2.14 ± 0.62(1–7)
Time to remove the abdominal drainage tube	5.30 ± 1.89(2–14)
Postopertaive hospital stay(days)	7.82 ± 1.91(4–16)
Perioperative deaths	1(0.7%)

Patients were encouraged to achieve early ambulation on postoperative day 1. The day on which each patient first left their bed is shown in Table 2. Early ambulation after laparoscopic surgery was achievable due to the absence of a long incision in the abdominal wall and minimal postoperative pain. The intraperitoneal drain tube was removed when the drainage fluid was limpid without any suggestion of internal bleeding and the total amount of ascites was <500 ml. Anticoagulation treatment was routinely implemented to prevent postoperative portal venous thrombosis. Anticoagulation therapy usually comprised enoxaparin administration due to its efficacy in treating and preventing portal vein thrombosis in cirrhotic patients (16).

Postoperative complications are exhibited in Table 3. The most common complication was acute portal vein system thrombosis, which occurred in 10 patients (6.85%), but did not progress to severe symptomatic portal venous thrombosis. The only perioperative death was related to esophagus variceal hemorrhage. A full review of this patient's clinical data suggested that this fatal postoperative bleeding event may have occurred due to multiple preoperative endoscopic sclerotherapies that formed extensive collateral varices surrounding the gastric fundus and extending to the middle esophagus; these varices impeded the routine exposure of the esophagus.

**Table 3:** Complications during hospitalization and PVT in follow-up (*n* = 146).

Complications	Value (*n*)	Death (*n*)
Intraperitoneal bleeding	**5**(3.42%)	
Upper gastrointestinal hemorrhage	**2**(1.37%)	1
Intractable ascites	**2**(1.37%)	
Hepatic encephalopathy	**0**	
Hepatorenal syndrome	**0**	
Gastric fistula	**0**	
Pancreatic fistula	**0**	
Incision infection	**0**	
Intra-abdominal infection	**1** (0.7%)	
Acute portal vein thrombosis	**10**(6.85%)	
Portal vein thrombosis in follow-up	**39**(26.71%)	

### Postoperative outcomes

One of the major concerns postoperatively is rebleeding, which is an important prognostic indicator. We also focused on the patient survival rate. Patients were followed up *via* telephone or clinical visit throughout the first year postoperatively. Ten patients were lost to follow-up, giving a follow-up loss rate of 6.9%. A total of 82 patients were followed up for 3 years postoperatively. The 1 to 3 year postoperative outcomes are exhibited in Table 4. One-hundred-and-thirty-five patients reached the endpoint of 1 year postoperatively; 11 patients (8.15%) experienced clinical relapse of rebleeding and two patients (1.48%) died in the first year. Ninety-six patients reached the endpoint of 2 years postoperatively; the cumulative rebleeding rate was 11.85% (16 patients), and one patient died in the second year. Eighty-two patients reached the 3-year observation endpoint; the cumulative rebleeding rate was 13.33% (18 patients) and the cumulative 3-year mortality rate was 3.66% (three patients). One rebleeding event and one more death occurred more than 3 years postoperatively. In total, 19 patients experienced a rebleeding event and four patients died.

**Table 4:** Rebleeding rate and mortality rate in follow-up (*n* = 135).

Postoperative time	Value (*n*)	Percentage
Re-bleeding rate		
1-year re-bleeding	11	8.15%
2-years re-bleeding	16	11.85%
3-years re-bleeding	18	13.33%
>3 year re bleeding	1	
Total	19	14.07%
Mortality		
1-year mortality	2	1.48%
2-years mortality	3	2.22%
3-years mortality	3	2.22%
>3 year mortality	1	
Total	4	2.96%

### Postoperative hemodynamic and endoscopic changes

Splenectomy and devascularization result in many hemodynamic changes that are rarely mentioned. We performed a self-controlled observational study using ultrasonic hemodynamic evaluation by measuring the pre- versus postoperative diameter, flow velocity, and blood flow of the vessels. Due to incomplete ultrasonic reports and insufficient out-patient data, we only enrolled those with ultrasonic hemodynamic information for more than 1 month postoperatively. Although the number of effective cases varied among the assessed variables, our results still indicated marked changes in the liver accessory vessels, including the portal vein system and hepatic artery, as exhibited in Table 5. There were significant postoperative decreases in the portal vein diameter (1.49 ± 0.23 cm versus 1.29 ± 0.27 cm), flow velocity (18.43 ± 5.40 cm/s versus 14.36 ± 3.64 cm/s), and flow amount (1,859.06 ± 678.42 ml/min versus 1,061.55 ± 481.85 ml/min) (*P* < 0.05). The splenic vein behind the pancreas was often fully blocked by thrombosis or decreased in diameter (1.20 ± 0.27 cm versus 0.79 ± 0.27 cm). In contrast, the hepatic artery showed significant postoperative increases in diameter (0.27 ± 0.05 cm versus 0.34 ± 0.08 cm), flow velocity (26.95 ± 10.82 cm/s versus 45.00 ± 13.40 cm/s), and flow amount (99.38 ± 49.34 ml/min versus 281.91 ± 171.76 ml/min) (*P* < 0.05). Interestingly, the superior mesenteric vein showed significant postoperative decreases in diameter (1.03 ± 0.19 cm versus 0.85 ± 0.17 cm) and flow amount (790.66 ± 372.20 ml/min versus 562.48 ± 235.98 ml/min) (*P* < 0.05), but the flow velocity (15.90 ± 5.40 cm/s versus 15.39 ± 3.95 cm/s) remained stable (*P* = 0.586). Surgery resulted in a decrease in blood flow through the portal vein system to reduce the vascular pressure, while the increase in hepatic artery flow improved the hepatic oxygen supply. The endoscopy examination showed that the red color sign after operation was significantly lower than that before operation (*P* = 0.012), though the degree and the classification of varicose veins after operation were not significantly improved. That may be related to the varices still connected with small blood vessels in the stomach even the root of varicose has been cut off. And endoscopic follow-up treatment may further lower the risk of upper intestinal bleeding but more need to be explored. However, the decrease of red color sign still strongly suggested that the risk of upper gastrointestinal bleeding after operation was reduced. This is consistent with the improvement of portal hemodynamics.

**Table 5:** Hemodynamic and endoscopic changes before and after operation (*n* = 145).

		Before operation	After operation	D-value	V	*P*-value
Portal vein	The diameter (cm)	1.49 ± 0.23	1.29 ± 0.27	0.20 ± 0.24	95	<0.001
	Flow velocity (cm/s)	18.43 ± 5.40	14.36 ± 3.64	4.07 ± 7.07	51	<0.001
	Flow amount (ml/min)	1,859.06 ± 678.42	1,061.55 ± 481.85	797.51 ± 804.20	51	<0.001
Splenic vein	The diameter (cm)	1.20 ± 0.27	0.79 ± 0.27	0.41 ± 0.31	74	<0.001
Superior Mesenteric vein	The diameter (cm)	1.03 ± 0.19	0.85 ± 0.17	0.18 ± 0.24	90	<0.001
	Flow velocity (cm/s)	15.90 ± 5.40	15.39 ± 3.95	0.51 ± 6.61	50	0.586
	Flow amount (ml/min)	790.66 ± 372.20	562.48 ± 235.98	228.19 ± 458.09	50	0.001
Hepatic artery	The diameter (cm)	0.27 ± 0.05	0.34 ± 0.08	−0.08 ± 0.10	36	<0.001
	Flow velocity (cm/s)	26.95 ± 10.82	45.00 ± 13.40	−18.05 ± 17.29	19	<0.001
	Flow amount (ml/min)	99.38 ± 49.34	281.91 ± 171.76	−182.53 ± 186.40	19	0.001
Endoscopy	Red color sign			6.269	1	0.012
	Yes	114 (78.1%)	94 (64.8%)			
	No	32 (21.9%)	51 (35.2%)			
	Gastroesophageal varices			3.161	1	0.075
	<5 mm	18 (12.3%)	29 (20.0%)			
	>5 mm	128 (87.7%)	116 (80.0%)			
	classification			3.671	1	0.055
	Mild to moderate	40 (27.4%)	55 (37.9%)			
	Severe	106 (72.6%)	90 (62.1%)			

### Postoperative liver function improvement and liver regeneration

**Figure 4:**
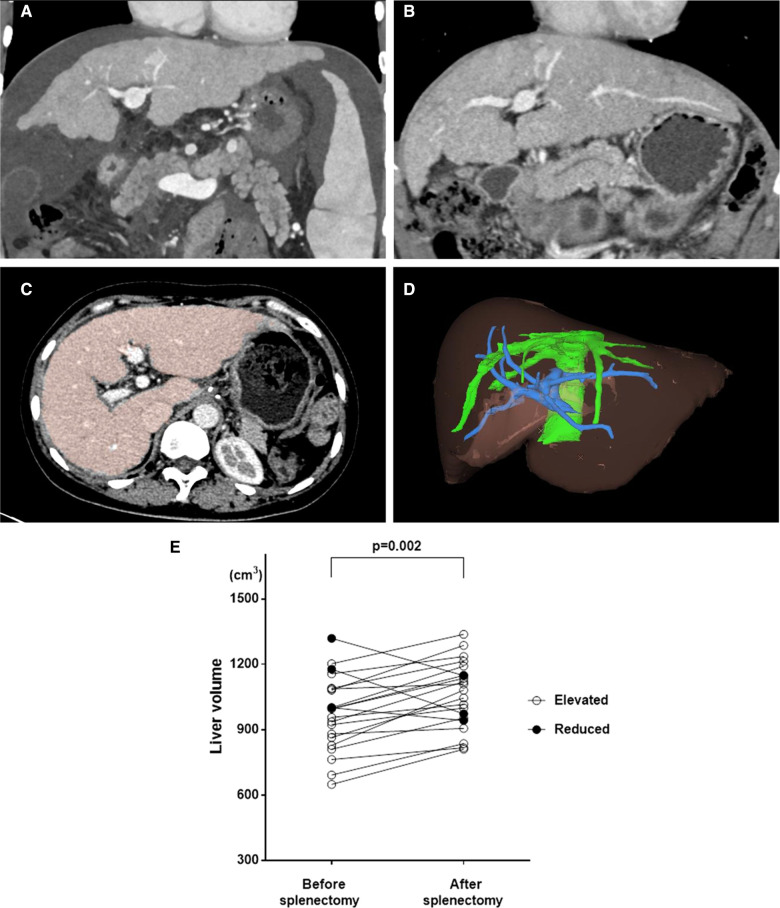
Liver regeneration were observed in many patients after surgery, we applied The Myrian® XP-Liver Work Environment (Myrian, Intrasense, France) to measure the liver volume. (**A**) CT image before surgery showed liver cirrhosis and reduced liver volume; (**B**) CT image after surgery on same layer exhibited increased liver volume which indicated liver regeneration after surgery; (**C**) The liver was stained in color to measure liver volume; (**D**) A 3-D image of liver was built to calculate the accurate liver volume; (**E**) The significant increasing of Liver volume were observed in 19 of 21 patients with integrity CT data of before and 3 months after surgery.

Pre- and postoperative CT data had been recorded in our department since 2018. We performed a 3D liver volume evaluation of the 21 patients who had both preoperative and 3-month postoperative CT data available (Figure 4). Nineteen of 21 patients had a significantly increased liver volume postoperatively, indicating liver regeneration (Figure 4E). We also compared the liver function at the two timepoints when CT was performed. As shown in Table 6, the Child-Pugh class and ICG score did not differ between the two timepoints in each patient, but the MELD score significantly decreased, indicating specific improvement in the liver function reserve. This result provided important evidence that surgery benefited patients with PHT. The aim of surgery is to treat or prevent bleeding events in patients with PHT. If surgery is confirmed to relieve cirrhosis and promote liver regeneration, surgery should be recognized as not just a symptomatic treatment but an etiological treatment.

**Table 6:** Liver function improvements and liver volume increasement (*n* = 21).

	Preoperation	Postoperation	D-value	*P*-value
Child-Pugh	6.29 ± 0.64	6.19 ± 0.40	0.095 ± 0.70	0.54
Child-Pugh class				
A	15(71.4%)	17(81.0%)		0.719
B	6(28.6%)	4(19.0%)		
MELD score	9.62 ± 1.99	7.78 ± 1.50	1.84 ± 2.08	<0.001
Liver volume (cm^3^)	971.76 ± 171.54	1,061.90 ± 151.82	−90.14 ± 117.31	0.002

## Discussion

Liver cirrhosis and subsequent PHT result in great burdens on the Chinese healthcare system. Although liver transplantation is the only cure, most patients cannot receive this treatment due to a lack of donors (official Chinese reports state that there were only 6,302 organ donations after death in 2019) and financial problems. All other treatments except for liver transplantation are considered non-etiological treatments. It remains unclear how best to relieve the severity of cirrhosis and improve the survival of patients with PHT. Surgery (including shunt and no-shunt procedures) has been used to treat PHT for more than 60 years (17), and is still widely accepted and applied in China, especially in our department (18). The most common surgical procedure in China is splenectomy plus devascularization, while shunt operations are also used in some institutions. The potential survival benefit of surgery has been reported in an early randomized clinical trial (8). The survival benefits of surgical shunt procedures are controversial, as the fundamental principles of all shunt procedures are similar, and the therapeutic effects of surgical shunt operations are similar to or better than TIPS in select patients (19). Our department has applied devascularization and shunt operations to treat PHT for decades. Since laparoscopy was first introduced in splenic surgery, we considered that laparoscopic techniques could make PHT surgery less invasive, safer, and more effective. Our department has performed laparoscopic splenectomy and devascularization since 2010. Two years later, this surgical procedure was sufficiently mature to become a routine operation. The individualized evaluation and operation for PHT described in the present study was formed in 2013 and has been routinely used since. This is the first report of an individualized evaluation and surgical procedure for patients with PHT.

The traditional evaluation of patients with PHT focused on the risk of rebleeding, as rebleeding often leads to mortality without effective treatment (7). However, the appropriate evaluation of those without a clear history of upper gastrointestinal hemorrhage is still debatable. We believe that the first bleeding event is far more important for patients with PHT, as the event has a high mortality rate within 6 weeks and commonly results in decreased liver function (20). For patients with PHT, decreased liver function leads to fatal complications including hepatic encephalopathy and liver failure. It remains challenging to distinguish those with a high risk of first bleeding from the general PHT population. We believe that the combination of large gastroesophageal varices, red marks, and high HVPG (>12 mmHg) (21) suggest a high bleeding risk and the need for more active interventions like surgery. In our department, patients with PHT with these conditions undergo surgery to prevent the first bleeding event instead of waiting for it to happen.

We applied a individualized 3D remodeling evaluation for each patient to identify the major collaterals connecting the portal vein and varices. The devascularization procedure was based on the reorganization of gastric fundal varices as the primary cause of gastrointestinal hemorrhage in PHT (11). It is widely accepted that the major collateral linking the varices and the portal vein is the left gastric vein (vena coronaria). The left gastric vein consists of two main branches (esophageal and gastric), which may be the primary drainage branch of gastroesophageal varices either alone or together. We also found that the primary feeding vessel of gastroesophageal varices in some patients was the posterior gastric vein and/or short gastric vein, which were previously ignored (Figure 1B). The individualized and precise devascularization procedure was planned in accordance with the 3D reconstructed model of the major collaterals for each patient. The enlarged spleen was always the biggest obstacle to the surgical procedure due to its localized mass effect. In our experience, ligating the splenic artery is the most important step before dissociating the connective tissue around the spleen; when the blood flow into the spleen was blocked, the size of the spleen shrunk and the perisplenic space appeared, making it easy to separate. The perioperative results proved that this surgical procedure was relatively safe in select patients with PHT. The adverse events of this surgical procedure were intraoperative conversions to open surgery due to uncontrolled blood loss in eight patients and unplanned reoperations in three patients. Care is needed during any surgical procedure in patients with PHT due to the high perioperative mortality rate accompanied by decompensated liver cirrhosis (22). To ensure surgical safety, the fast-open technique for open surgery remains important in patients with PHT. We believe that this surgical procedure is safe in experienced hands.

Portal vein thrombosis is a perioperative complication with a high incidence of 26.71%. However, in our experience, the initiation of anticoagulant therapy soon after surgery prevents portal vein thrombosis from becoming a problem in most patients; portal vein thrombosis is generally asymptomatic and relatively harmless unless occlusive portal vein thrombosis forms. In the present study, as early and continuous anticoagulant therapy was applied to all patients, no patients developed occlusive portal vein thrombosis. Thus, we recommend anticoagulation therapy to prevent occlusive portal vein thrombosis after surgery in patients with PHT.

The main endpoints in the present study were rebleeding events and mortality. These two outcomes can also be used to assess the therapeutic effects of any PHT treatment strategy. It remains controversial whether shunt procedures or devascularization are superior in treating PHT. The TIPS is the most widely applied shunt procedure. TIPS stops acute hemorrhage, but still has a cumulative rebleeding rate of 20%–30%, offers no survival benefit or positive impact on liver function (5, 19), and has a high post-treatment encephalopathy rate of 20%–70%. Despite the difference in treatment populations between surgery and TIPS, the surgical procedure reported in the present study exhibited very low cumulative rebleeding rates of 8.15% at 1 year, 11.85% at 2 years, and 13.33% at 3 years. The overall rebleeding rate in our study was similar to that reported in previous studies (23, 24), but a stratification of patients by follow-up time revealed that rebleeding events were experienced by 11 patients in the first year, but only five of 105 patients in the second year and two of 82 patients in the third year. This may be due to the surgical procedure focusing on the vessels outside rather than the varices inside the gastroesophageal tract. Thus, sequential endoscopic therapy may help lower the incidence of postoperative rebleeding.

The mortality rate in the present study was much lower than that reported for TIPS and similar surgery (24). Therefore, our surgical treatment may protect liver function and improve survival by removing the enlarged spleen as previously reported (25, 26). The splenomegaly contributes to almost 40%–60% of the blood supply of the portal veinous system. And therefore, splenectomy may be profit to decrease the portal inflow and pressure. The normalization of the shear stress of the portal pressure helps improve liver function. The postoperative hemodynamic changes may partly reveal the positive effects of surgery on the liver function of patients with PHT. The present surgical procedure decreased the diameter and blood flow amount of the portal vein, but increased the diameter and blood flow amount of the hepatic artery, which improved the oxygen supply to the liver.

The most interesting finding of the present study was the significant increase in liver volume in some patients after surgery, similarly to a study from Japan (27). Despite the possible effects of surgical treatment, splenectomy may interfere with the common pathological process through liver-spleen cross-talk in liver cirrhosis (28). Our results support the theory that surgical treatment including splenectomy may act as not only a symptomatic treatment by preventing hemorrhage from esophageal-fundic varices, but also an etiological treatment to improve liver function and relieve cirrhosis, and even promote liver regeneration. As PHT comprises extremely complex physical and physiological processes with disorder of multiple vasodilators (29), further study is needed to reveal the influence of surgical treatment in patients with PHT.

The limitation of the study is relatively small sample size retrospective study. The exploration of the relationship between endoscopy and 3D CT images based on large clinical samples may assess a new approach to the portal hypertension patients.

In conclusion, we introduced an individualized and precise total laparoscopic surgical procedure for PHT. We also summarized the preoperative evaluation, surgical procedure, perioperative complications, and short- and long-term outcomes. The limitation of this study is the small sample size, which may represent only a subset of patients with PHT. The present results need validation in a larger population or in other centers, but suggest that this operation is a safe, feasible, and effective surgical procedure. Our results also suggest that surgical treatment may not only act as an effective symptomatic treatment for PHT to prevent esophageal and gastric hemorrhage, but may also have a positive impact on long-term survival as an etiological treatment.

## Data Availability

The raw data supporting the conclusions of this article will be made available by the authors, without undue reservation.
